# Morphological and mechanical properties of flexible resilin joints on damselfly wings (*Rhinocypha* spp.)

**DOI:** 10.1371/journal.pone.0193147

**Published:** 2018-03-07

**Authors:** Mamat-Noorhidayah, Kenjiro Yazawa, Keiji Numata, Y. Norma-Rashid

**Affiliations:** 1 Institute of Biological Science, Faculty of Science, University of Malaya, Kuala Lumpur, Malaysia; 2 Enzyme Research Team, Biomass Engineering Research Division, RIKEN Center for Sustainable Resource Science, Wako-shi, Saitama, Japan; Brown University, UNITED STATES

## Abstract

Resilin functions as an elastic spring that demonstrates extraordinary extensibility and elasticity. Here we use combined techniques, laser scanning confocal microscopy (LSCM) and scanning electron microscopy (SEM) to illuminate the structure and study the function of wing flexibility in damselflies, focusing on the genus *Rhinocypha*. Morphological studies using LSCM and SEM revealed that resilin patches and cuticular spikes were widespread along the longitudinal veins on both dorsal and ventral wing surfaces. Nanoindentation was performed by using atomic force microscopy (AFM), where the wing samples were divided into three sections (membrane of the wing, mobile and immobile joints). The resulting topographic images revealed the presence of various sizes of nanostructures for all sample sections. The elasticity range values were: membrane (0.04 to 0.16 GPa), mobile joint (1.1 to 2.0 GPa) and immobile joint (1.8 to 6.0 GPa). The elastomeric and glycine-rich biopolymer, resilin was shown to be an important protein responsible for the elasticity and wing flexibility.

## Introduction

Insects were among the first animals that have been recognised to have unique structures of elastic proteins that assist in movements [1]. Insect wings, including those of dragonflies are complex mechanical structures. A study reported a venous system for dragonfly wings, mainly composed of veins and membranes, possessing both stiff and flexible materials [2]. Wang et al [3] further reported the wing veins of complex sandwich structure of chitinous shells and a protein layer containing fibrils that further enhance the capability to absorb mechanical energy [4]. This hierarchical composite structure is said to be common in biological materials [5, 6]. While the deformations of the wing in Odonata are known to be contributed by a one-way hinge that allows the free movements in the upstroke and restrains the displacements in the downstroke [7], it is influenced by a number of criterion over the wing, one of the most effective elements are vein micro joints in contrast to others [8, 9, 10].

The main focus of this study is on the flexible element that has been found on the wings of damselflies, called resilin. Resilin is the rubber-like protein found in specialized regions of the cuticle of most insects that gives low stiffness, high strain and efficient energy storage [11–13] that functions in insect flight [14, 15]. Weis-Fogh [14] first described resilin from the flight systems of locusts and dragonflies, it was described to be similar to swollen isotropic rubber, but its elastic behaviour is unlike any other natural or synthetic polymer. Additionally, resilin was shown to have remarkable mechanical properties where it is two decades higher than for elastin, suggesting that resilin is a more mobile bio-polymer [16].

Numerous authors found that irrespective of its small size, the resilin-filled joints played a role in bending shapes and for the whole flexibility of the wing [17, 18]. Additionally, a study reported on the automatic performances of passive wing movements in odonates were made possible by the distribution pattern of resilin [15]. The presence of resilin in some vein joints of odonate provided flexibility and preventing stress concentrations, besides functioning as a damper and stretchable component [9, 15, 19].

A number of findings had discussed the structure and mechanical properties of the membranes, alongside with the venations of the insect wings [20–23] as well as on flight aerodynamics [24–30]. Although the current understanding increased on the role of wing structural elements and mechanical properties, however, the precise function of the individual elements and their components are still vague. Over the past 16 years, scientific notion of insect flight has been substantively transformed by the presence of new experimental techniques from measuring aerodynamics of flight, to the actions of actin and myosin proteins in the muscles [31]. Nevertheless, a systematic study on individual wing components are needed to further understand the effect of each element in an efficient approach, such as that presented here in this study. Previous work has concentrated on samples from dragonflies (Sub-order: Anisoptera), here we report the first comprehensive investigation on the microjoint wing properties in the suborder Zygoptera that shows the potential of combining techniques of laser scanning confocal microscopy (LSCM), scanning electron microscopy (SEM), atomic force microscopy (AFM) to investigate resilin elasticity and analyse the protein components.

## Materials and methods

### Collection of damselfly species

Adult damselflies, *Rhinocypha* spp. (Odonata: Zygoptera) were collected within the Peninsular Malaysia in 2015, from Sungai Gabai Waterfall and Teladas Waterfall by the permission of Forestry Department Peninsular Malaysia (Permit Number: JH/100 Jld.7 (12)). Methods of sampling and preservation of Odonata were based on previously described standard method [32, 33]. The species collected were identified as *Rhinocypha fenestrella*, *Rhinocypha perforata* and *Rhinocypha biforata*. The specific numbers of samples utilized were stated within the specific section of investigations. The forewings and hindwings from preserved specimens were removed from the body and were used in this study. Life specimens were not used since according to Xiao et al [34], there are no differences between the material properties of the live and dead dragonflies.

### Laser scanning confocal microscopy (LSCM)

The forewing of the samples were dried, mounted between two coverslips, and observed using a ZeissLSM 700 laser scanning confocal microscope in wavelengths of UV Bands (excitation 405nm, emission 400-420nm). Resilin was reported with auto fluorescence at a narrow band of wavelengths ~420nm [1, 17, 35, 36]. Wing veins were examined for the presence of resilin, which appears as a deep blue colour under UV excitation on both the dorsal and ventral sides. Images were captured with a specific digital camera AxioCam MRm up to a magnification of 40x, and detailed joint-by-joint mapping of resilin on the wings of the *Rhinocypha* spp. were conducted. A given longitudinal vein was scored as “present” with resilin when indicated by visible blue fluorescence and “absent” for vice versa. Veins were identified using homology system developed by Riek and Kukalova-Peck [37]. It should be mentioned here that there was a limitation in the mapping of resilin since there was a variation in the size (strong to weak) of resilin patches that were subjected fluorescence intensity. Thus, the presence and absence of the resilin at the specific veins (using the homology system) were more focused and thus scored here.

### Scanning electron microscopy (SEM)

After imaging by laser scanning confocal microscopy, the forewings were coated with gold-palladium and examined using a JOEL JCM-6000 NeoScope Benchtop scanning electron microscopy (SEM) at accelerating voltage of 5kV. The samples were mounted using carbon tape and examined for the presence of cuticular spikes in close proximity to the vein joint on the dorsal and ventral. Subsequently, a detailed joint-by-joint mapping of the spikes was done.

### Atomic force microscopy (AFM)

For this purpose the forewings were dissected into sections comprised of: wing membrane and veins which were divided into mobile and immobile joints. These samples were characterized using atomic force microscopy (AFM), where the wing parts were mounted onto a platform using a double-sided tape. The samples were observed and the images were recorded by using a Scanning Probe Microscope (AFM5300E, Hitachi High-Tech Science Corporation, Japan), at room temperature using a silicon cantilever type probe (SI-DF3, Seiko). The cantilever was set to a radius tip curvature of 10nm, with a spring constant of 1.7N/m and frequencies of 28kHz. The samples were scanned using tapping mode AFM with size of 1*μ*m x 1*μ*m. Three samples were used for each species of *Rhinocypha* spp., and the force curves from 10 different points at each section (wing membrane, mobile and immobile joints) were recorded from each sample for further elasticity measurements. The average values and standard deviations from the 30 different points at each section for the three species were used as the modulus values of each section of the wings. Prior to this, a force curve of a glass substrate was recorded as a reference.

### Mechanical properties–elasticity measurements with AFM

Elasticity or Young’s Modulus values were generated by analyzing the force curves according to the Hertz Model using parabolic tip geometry [38]. The loading force is defined as:

F=4Rc3E1−ν2δ32,

where *F* is the force, *Rc* is the radius of tip curvature, *E* is the elastic modulus, *ν* is the Poisson’s ratio and *δ* is the indentation depth. Afterwards, *E*-value was derived by fitting the indentation curve using Kaleida graph software.

Besides, to compare the Young’s modulus values for membrane of the wing, mobile joint and immobile joint among the three species, Kruskal Wallis test as a nonparametric test was applied due to non-normal distribution of samples data and was calculated using IBM SPSS statistic 24 for Windows (SPSS Inc., Chicago).

### Protein analysis of the wing samples

In this study, 16 dried wings were used for each *Rhinocypha* species comprised of a pair of both forewings and hindwings obtained from four samples of each species. The wing samples were dried and grounded to fine powder Eight milligrams of the powder was hydrolyzed in 8 M urea, 100 mM NaH_2_PO_4_, 10 nm Tris = pH 7.4, and stirred overnight. The lysate was centrifuged (10000 rpm, 90 min, r.t.) to transfer the supernatant into membrane filter (100–500 Da MWCO) for dialysis process for 5 days. Next, the hydrolysates were freeze-dried under a vacuum using an EYELA Rotary Vacuum Evaporator. Amino acid was analyzed by the Research Resources Centre of RIKEN Brain Science Institute using a Hitachi Amino Acid Analyzer (Biochrom) and about 0.7 mg/100 uL, 0.8 mg/100uL and 09 mg/100 uL of filtrate was taken for amino acid analysis for *R*.*biforata*, *R*. *perforata* and *R*. *fenestrella* respectively.

## Results

### Wing joint morphology

**Fig 1:**
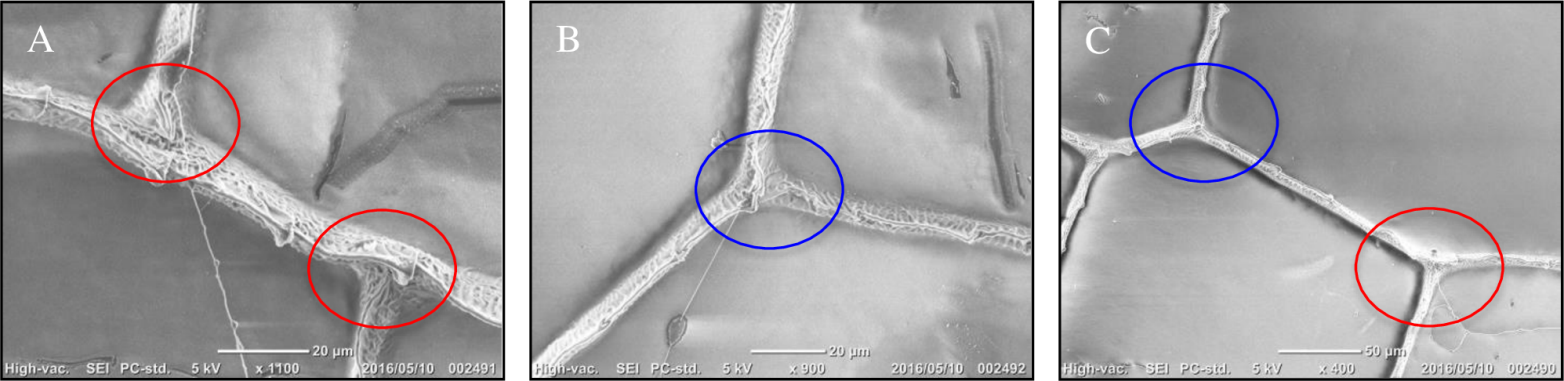
Scanning electron microscope (SEM) of the two types of vein joints. Red circle indicated the mobile joint while blue circle, the immobile joint. The illustrated Fig 1(A): Mobile joint at the cross vein attached with the longitudinal vein. (B): Immobile joint at the trailing edge of the wing. (C): Two types of the vein joint in the region of the MP(-).

Initial scanning electron microscopy (SEM) on the wings of *R*. *fenestrella*, *R*. *biforata* and *R*. *perforata* confirmed that there were two main types of joints; mobile and immobile (Fig 1). The mobile joints (Fig 1A and 1C) were disjointed to the longitudinal veins while the immobile joints (Fig 1B and 1C) were resolutely connected to the longitudinal veins.

**Fig 2:**
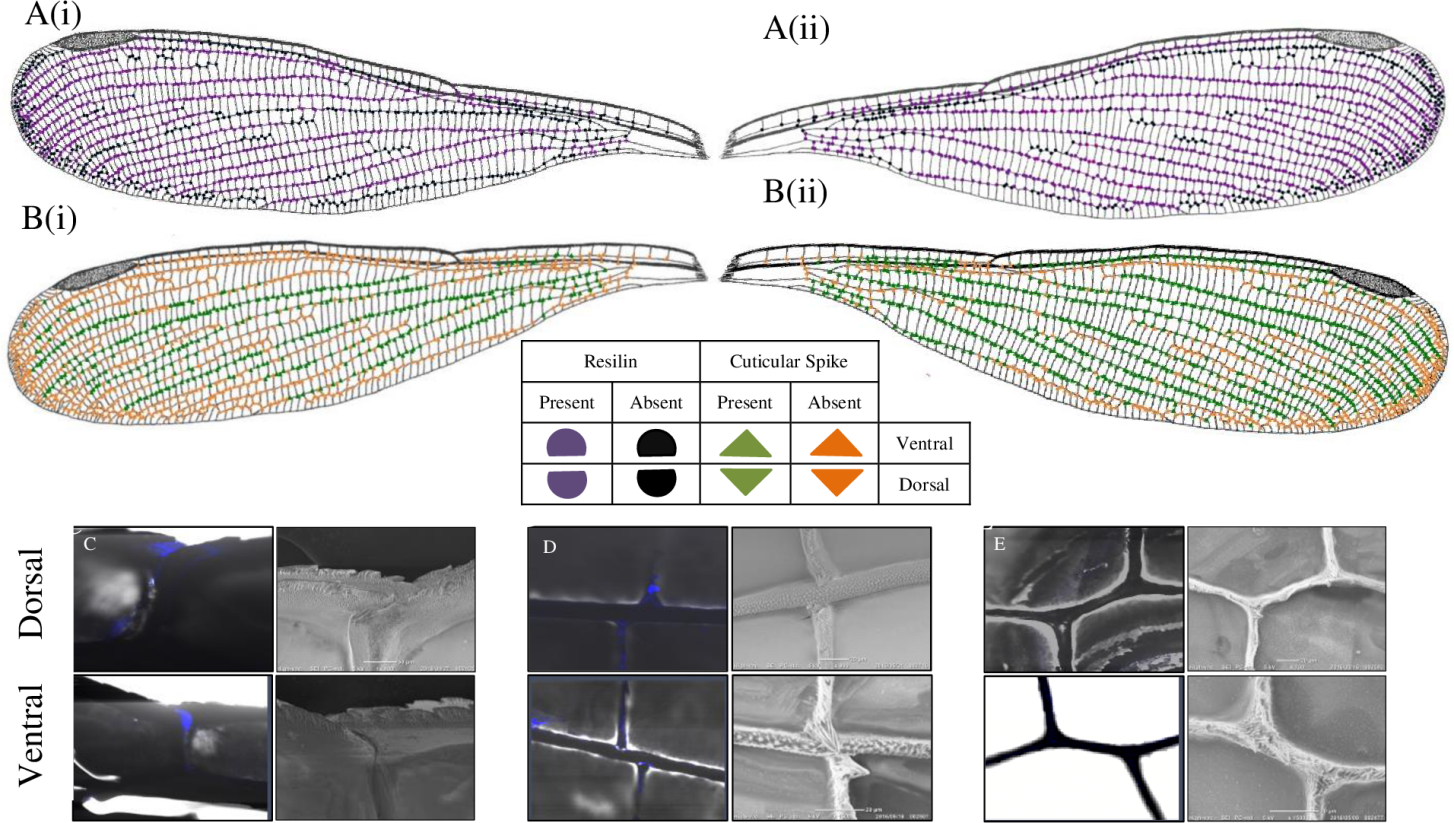
Distribution of resilin and spikes in the vein joints of *R*. *fenestrella* wing. A(i) and B(i) indicated dorsal side of the wing, while A(ii) and B(ii) indicated the ventral surface of the forewing. C-D: FM (right) and SEM (left) images at the selected joints for dorsal (upper) and ventral (below) surfaces. (C): Large resilin patches on both dorsal and ventral surfaces. (D): Resilin patches at the mobile joint. (E): No resilin patch at the immobile joint.

**Fig 3:**
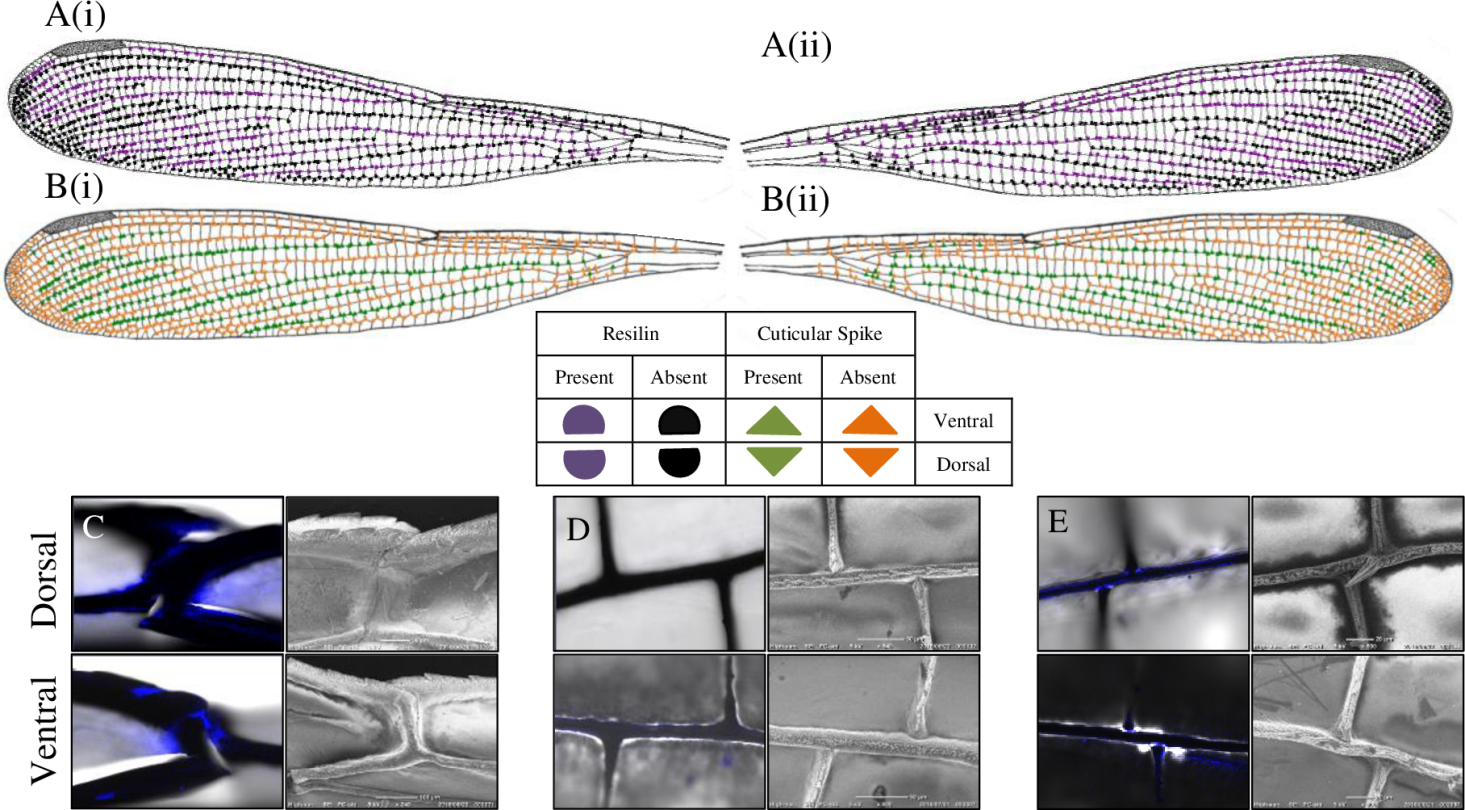
Distribution of resilin and spikes in the vein joints of *R*. *perforata* wing. A(i) and B(i) indicated dorsal surface of the wing, while A(ii) and B(ii) indicated the ventral surface of the forewing. C-D: FM (right) and SEM (left) images at the selected joints for dorsal (upper) and ventral (below) surfaces. (C): Large resilin patch in both dorsal and ventral surface. (D): No resilin patches at the ventral surface. (E): Resilin patches at the mobile joint for both dorsal and ventral surfaces.

**Fig 4:**
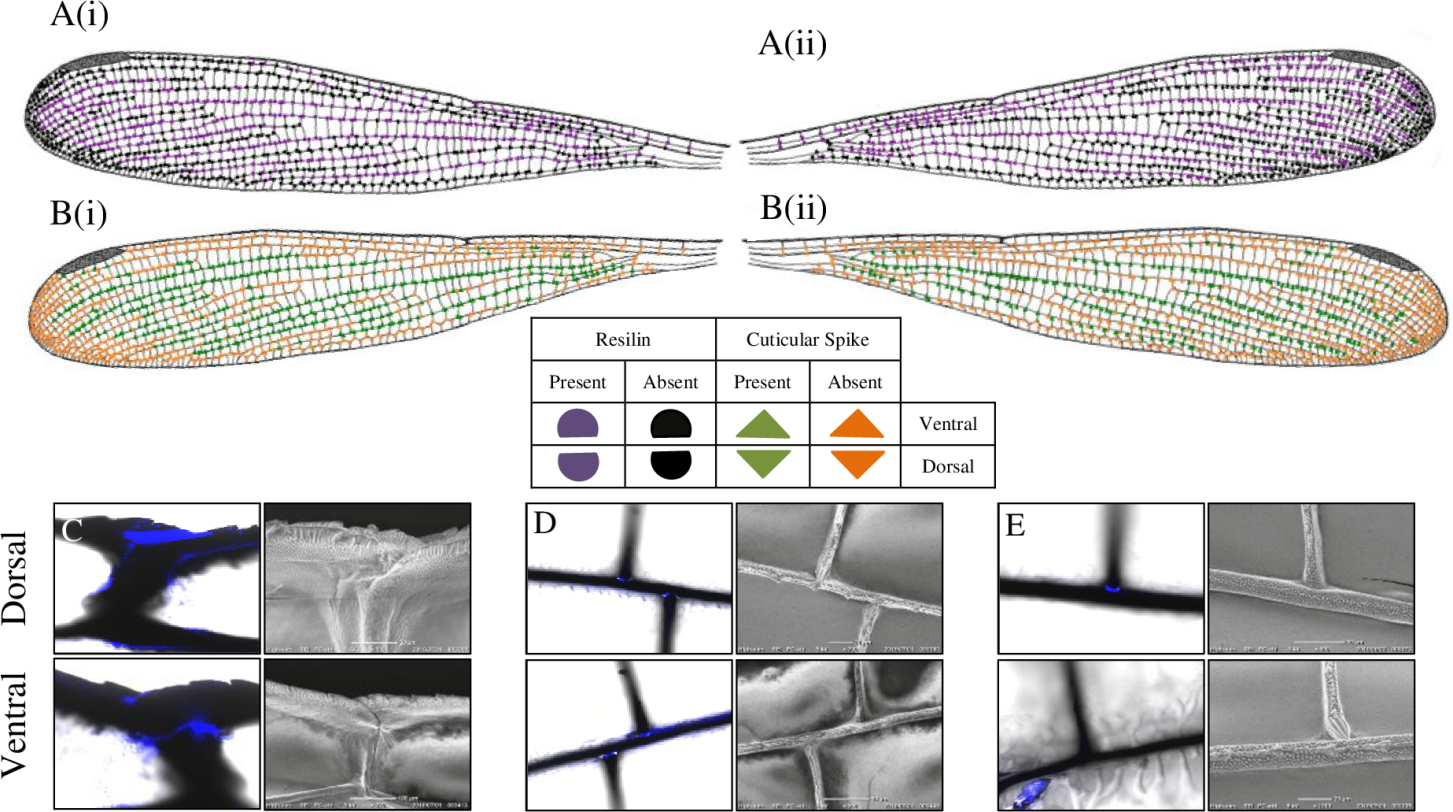
Distribution of resilin and spikes in the vein joints of *R*. *biforata* wing. A(i) and B(i) indicated dorsal surface of the wing, while A(ii) and B(ii) indicated the ventral surface of the forewing. C-D: FM (right) and SEM (left) images at the selected joint for dorsal (upper) and ventral (below) surface. (C): Large resilin patches on both dorsal and ventral surfaces. (D): Resilin patches on both dorsal and ventral surface. (E): Only resilin patch on ventral surface.

Laser Scanning Confocal Microscopy (LSCM) in the UV band revealed the presence of the blue-fluorescing material in the vein-joints, known as resilin, which was confirmed by the resilin-specific custom filter set (Figs 2–4). Resilin was found mostly in the mobile joints where cross veins met longitudinal veins (Figs 2D, 3E, 4D and 4E); whereas strong patches of resilin were found within the nodus for both dorsal and ventral surfaces (Figs 2C–4C).

**Fig 5:**
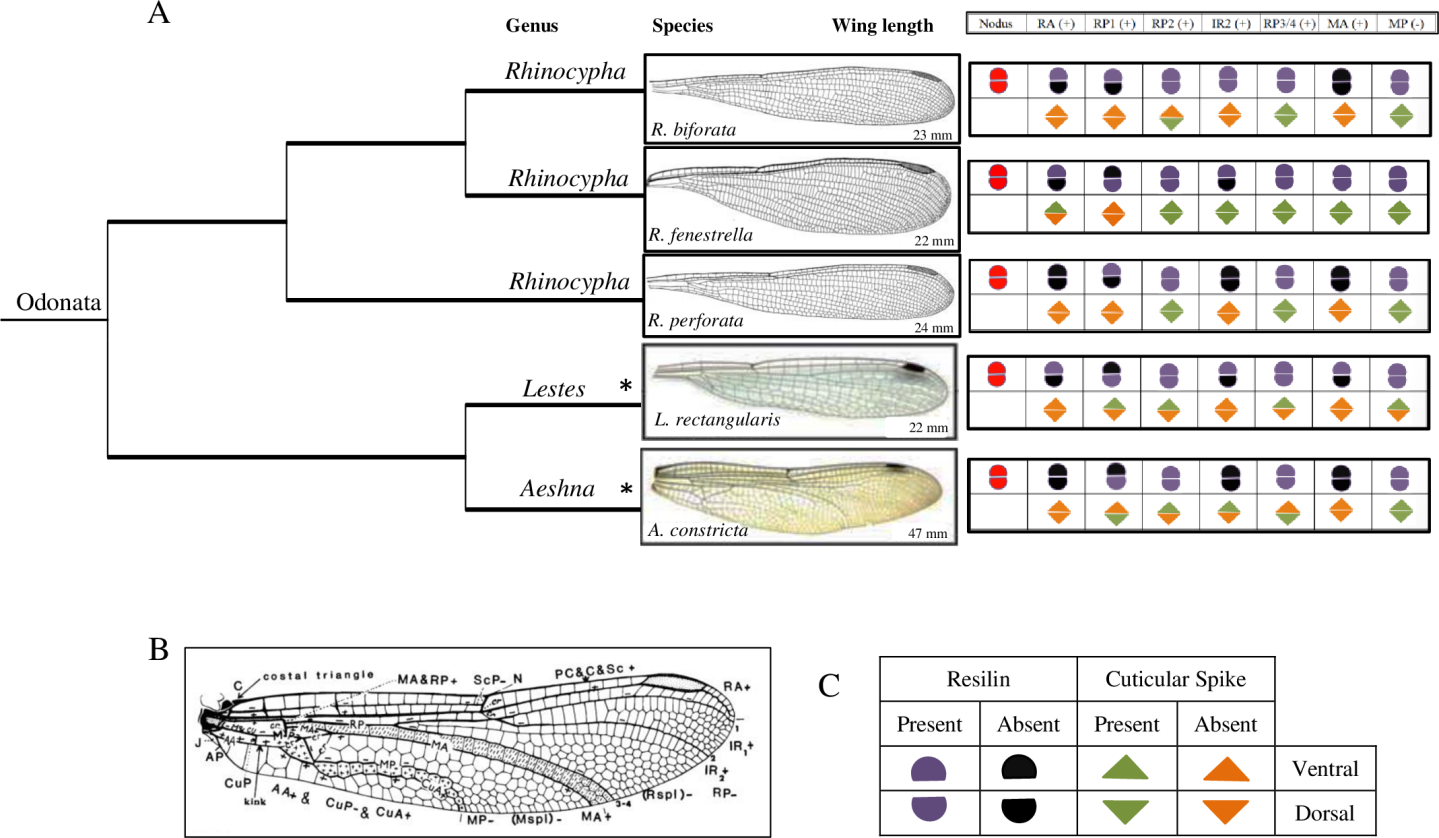
Summary of resilin and spikes mapping for the *Rhinocypha* group. (A) Phylogenetic comparison of resilin patches and spikes in damselflies. Branches show the relationship of taxa based on molecular, cytochrome oxidase c subunit 1 (COI) gene. (B) Nomenclature of wing structure according to Riek & Kukalova-Peck [37]. (C) Symbols used for resilin and spikes mapping. “Present” and “Absent” are marked at the vein that showed blue fluorescents and vice versa, and at the vicinity of vein joint for spikes. Findings from Donoughe et al [17]- were added to the figure, and these were indicated by asterisks (*).

Interestingly, the presence of resilin varied on the dorsal and ventral surfaces of specific joints revealing 4 distinct resilin distributions: 1) contained resilin only dorsally (e.g., Fig 4E), 2) only ventrally (e.g., Fig 3D), 3) present on both surfaces (e.g., Figs 2D, 3E and 4D) and 4) absent on both surfaces (e.g., Fig 2E). Overall, Fig 5 presents a summarised resilin mapping for the *Rhinocypha* group investigated in this current work. From Fig 5A, it is proposed that 3 trends existed: (1) nodus is constantly enriched with resilin for both dorsal and ventral surfaces (2) the dorsal longitudinal vein RA always withresilin present (3) both dorsal and ventral surfaces with the longitudinal veins RP2, RP3/4 and trailing edge vein MP with presence of resilin.

**Fig 6:**
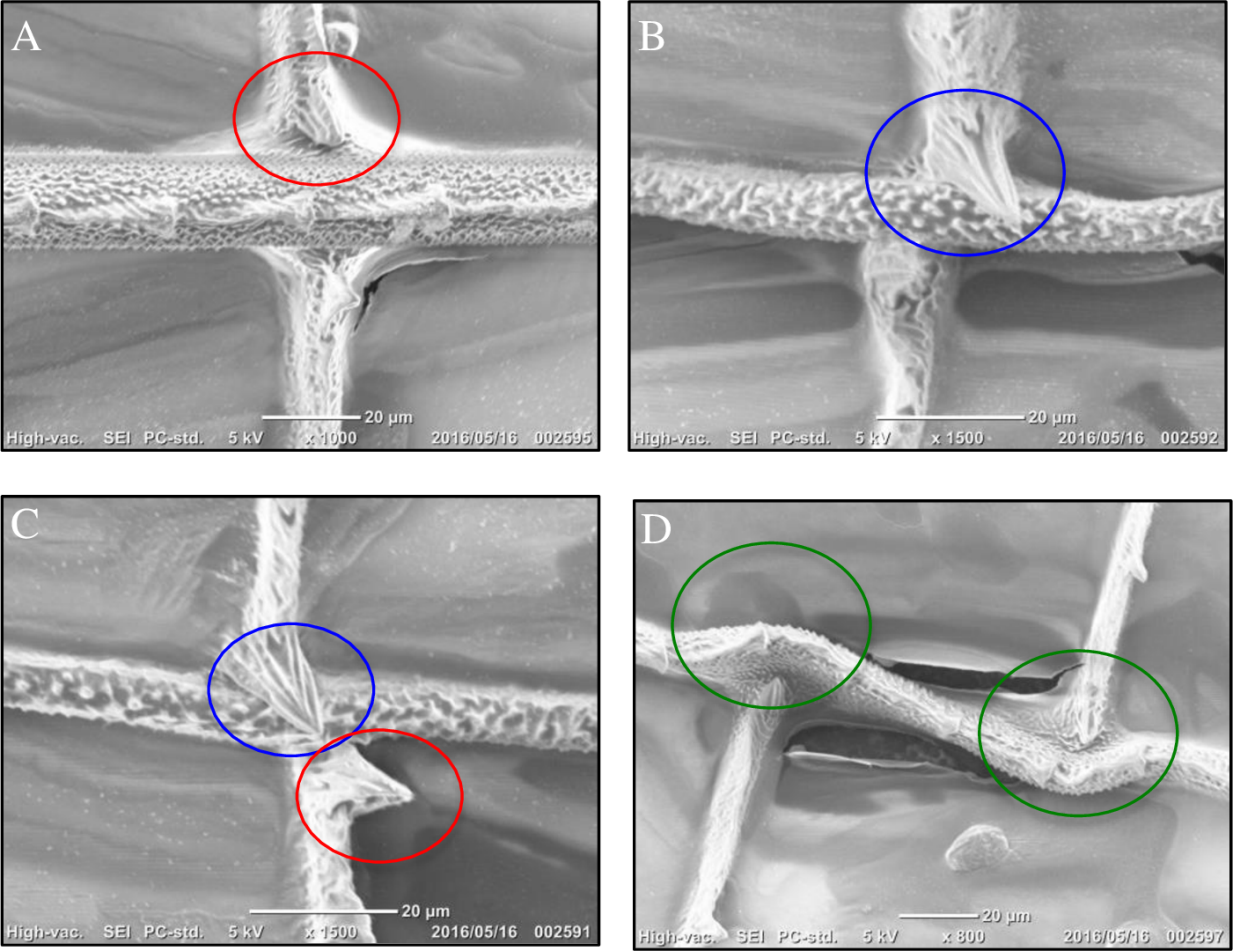
Relation of cuticular spikes with longitudinal vein by SEM imaging. (A) A spike upright protrusions pointing directly away from the wing surface. (B) A spike placed adjacent to the longitudinal vein. (C) Two types of spikes between cross vein and longitudinal vein. (D) Spikes orientated towards the longitudinal vein which impacted the wing veins.

Scanning electron micrographs (SEM) showed the position of spikes in relation to the longitudinal veins (Fig 6). Spikes were either upright protrusions pointing directly away from the wing surface (Fig 6A and 6C), placed adjacent to the longitudinal veins (Fig 6B and 6C) or orientated towards the longitudinal veins which impacted the wing veins (Fig 6D) by restricting the free movements of the joints.

The distribution of spikes at the wing vein joints was comparable to resilin, i.e: only dorsally (e.g., Figs 3D and 4D), only ventrally (e.g., Fig 4E), or on both dorsal and ventral surfaces (e.g., Fig 2D). Fig 5A illustrates the patterns of spike distribution true for all species: longitudinal vein RA, absence of spikes at the dorsal surface; longitudinal vein RP2 presence of spikes on the dorsal surface; spikes found at the trailing edge MP ventrally; and specific for *R*. *fenestrella*, longitudinal veins IR2 and MA have spikes on both dorsal and ventral surfaces. Generally, the results showed that spikes were widespread throughout the wing surfaces.

### Mechanical properties of the wing

In this study we investigated 3 sections of the wings: (1) membrane of the wing (2) mobile joint; and (3) immobile joint using the scanning electron microscopy (SEM) together with atomic force microscopy (AFM) to record the force curves across the samples, and analyse with the theoretical models to provide Young’s modulus value for the elasticity characteristic.

**Fig 7:**
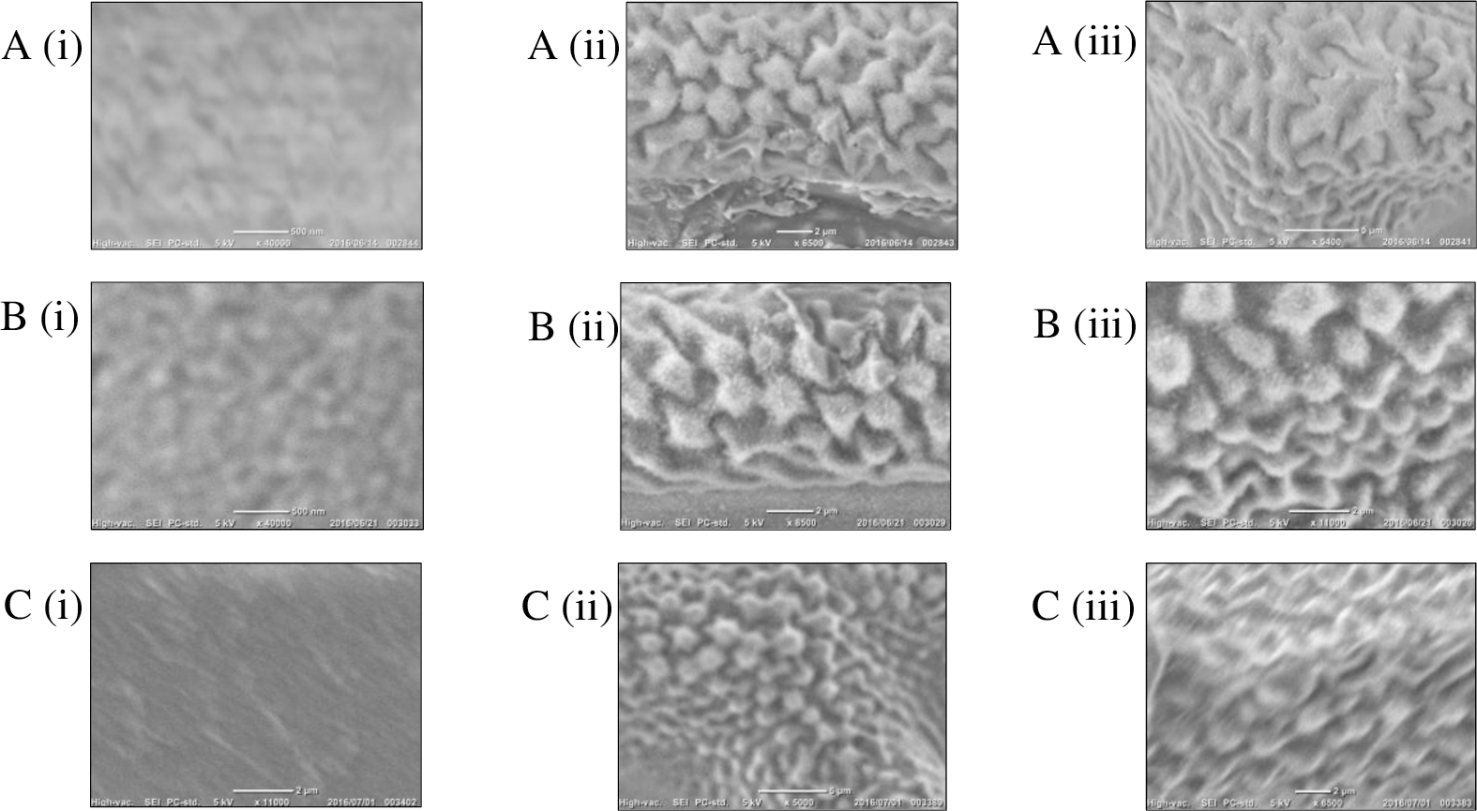
Surface of the wing images by SEM. (A); *R*. *fenestrella*, (B); *R*. *perforata*, (C); *R*. *biforata*. i–iii: the image for wing membrane, mobile joint and immobile joint respectively.

**Fig 8:**
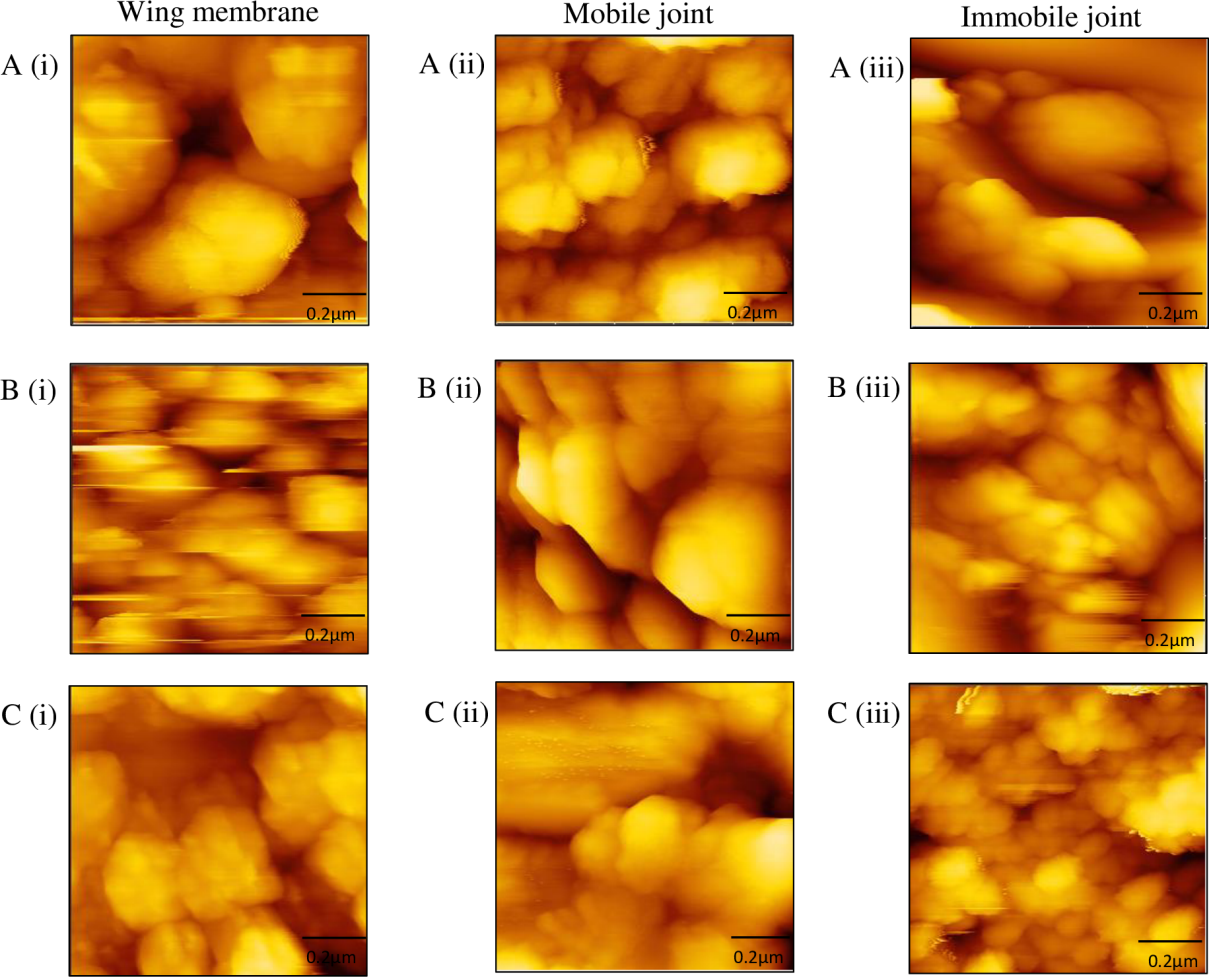
Morphologies of *Rhinocypha spp*. wings as observed under AFM. A(i), A(ii) and A(iii) potray the wing membrane, mobile joint and immobile joint of *R*. *fenestrella*; B(i), B(ii) and B(iii) indicated the wing membrane, mobile joint and immobile joint of *R*. *perforata*, while C(i), C(ii) and C(iii) indicated the wing membrane, mobile joint and immobile joint of *R*. *biforata* respectively.

Fig 7 shows the representative images of the membrane for each section of the wings of *Rhinocypha* spp. using the SEM, whereas Fig 8 shows representative topography images of the membrane for each section of each sample using the AFM in non-contact mode. Imaging from SEM revealed morphologies on the surfaces of the wings in the form of wavelike patterns (Fig 7C(i)), and additionally, there were globular nanostructures (e.g., Fig 7A(ii) and 7B(ii)) observed on the wing surfaces.

Force curves of the sample surfaces were recorded using a parabolic tip, and then it was analysed according to Hertz theory to give the quantitative data on sample elasticity (Young’s modulus) S1 Table. The Kruskal Wallis test was applied due to non-normal distribution of samples data as a nonparametric test to compare the Young’s modulus values for membrane of the wing, mobile joint and immobile joint among the three species. The results indicated that there were significant difference among species for membrane of the wing (KW = 35.433, p<0.001), mobile joint (KW = 7.5367, p = 0.024) and immobile joint (KW = 23.458, p<0.001).

Table 1 shows Young’s modulus values for the wing membranes, mobile and immobile joint sections for each of the three species of *Rhinocypha* and the Kruskal Wallis test pairwise comparison. The modulus values for the membrane of the wing, mobile joint and immobile joint of *R*. *fenestrella* were 0.04±0.04 GPa, 2.0±1.8 GPa and 6.0±5.6 GPa (mean ± standard deviations), respectively. Whereas the elasticity of the membrane of the wing, mobile joint and immobile joint of *R*. *perforata* was 0.04±0.03 GPa, 1.8±1.9 GPa and 2.1±2.8 GPa, as well 0.16±0.17 GPa, and the values;1.1±1.6 GPa and 1.8±1.8 GPa for *R*. *biforata*.

**Table 1:** Calculated values using the Young’s modulus formula for the 3 section wing samples from the 3 species, genus: *Rhinocypha*.

Species		Membrane of Wing		Mobile Joint		Immobile Joint	
	n	Mean±SD	Median (IQR)	Mean±SD	Median (IQR)	Mean±SD	Median (IQR)
***R*. *fenestrella***	30	0.043±0.039^b^	0.03 (0.03)	2.107±1.829^ab^	2.0 (3.90)	5.960±5.609^a^	4.0 (5.20)
***R*. *perforata***	30	0.036±0.038^b^	0.02 (0.03)	1.800±1.896^a^	1.0 (1.89)	2.073±2.783^b^	1.0 (1.65)
***R*. *biforata***	30	0.162±0.172^a^	0.10 (0.12)	1.054±1.609^b^	0.3 (0.83)	1.780±1.791^b^	1.0 (10.3)

Means with same letter are not statistically significant at 0.05 levels.

**Fig 9:**
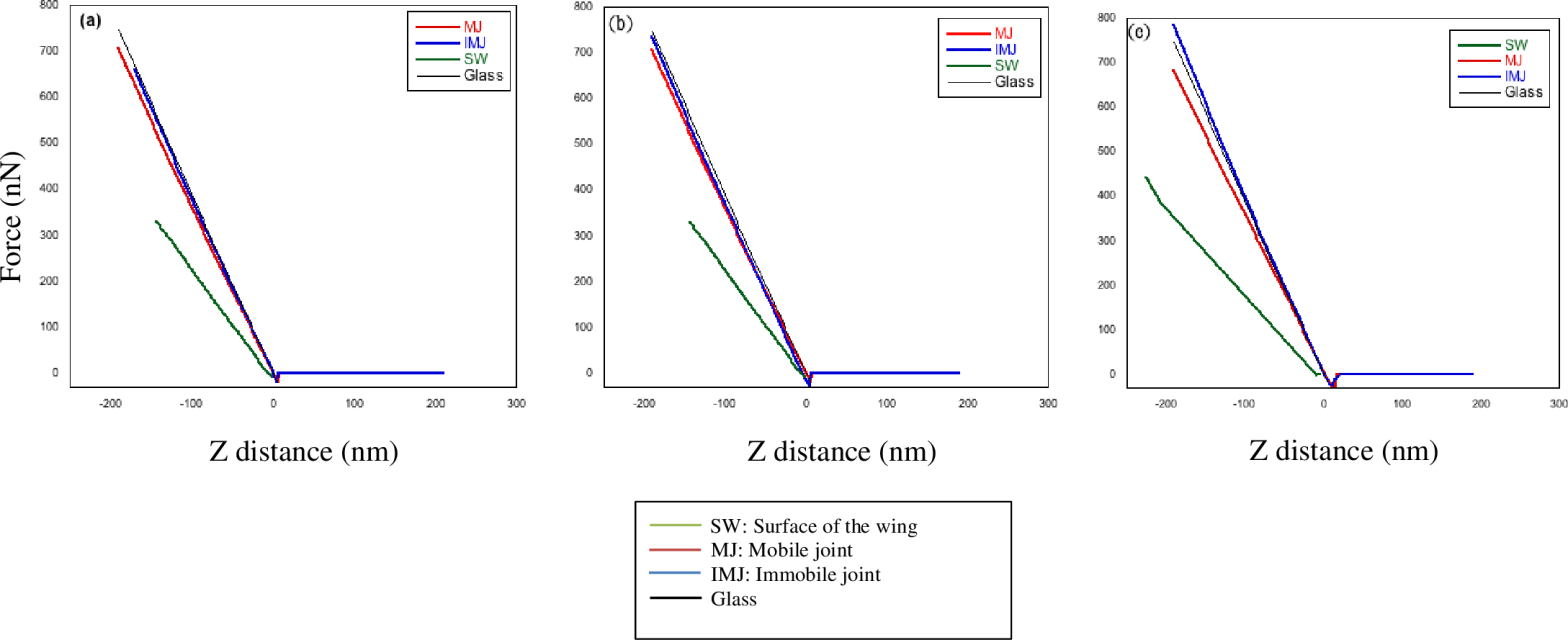
Force-displacement curve of the three sections of *Rhinocypha* spp. with glass as a reference. (a) represented the force-displacement curve for the species of R. *fenestrella*, (b) *R*. *perforata*, (c) *R*. *biforata* respectively.

**Fig 10:**
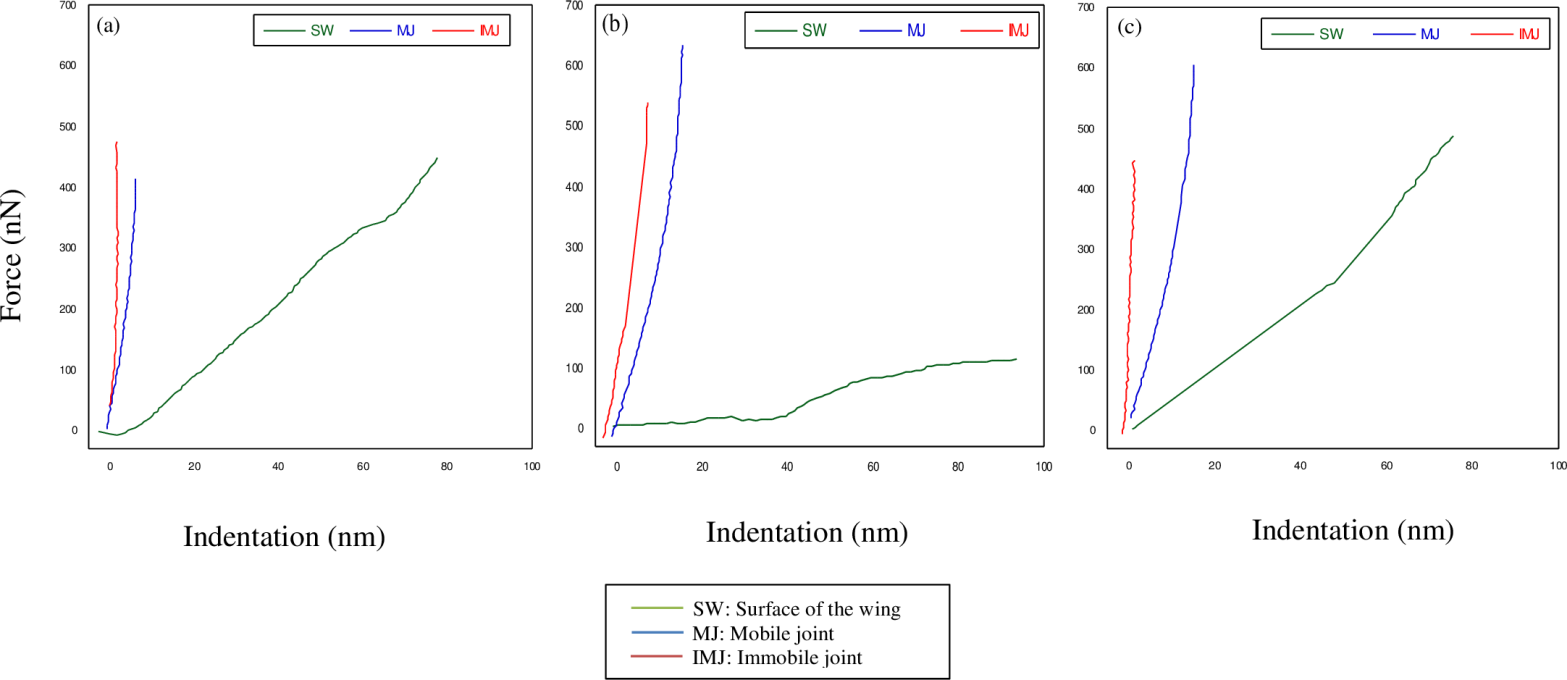
Force-indentation curve of the three sections of *Rhinocypha* spp. (a) represented the force-indentation curve for the species of R. *fenestrella*, (b) *R*. *perforata*, (c) *R*. *biforata* respectively.

Fig 9 shows a typical force-displacement curve of the three sections with glass sample as a standard reference for each species of *Rhinocypha*. For all the three species, the immobile joint had greater hard substance compared to both mobile joint and membrane of the wing. The force-indentation curve (Fig 10) for the three sections of the sample reinforced these results further, revealing descending order of stiffness; immobile joint > mobile joint > membrane of the wings for all the three species of *Rhinocypha*. Thus, these results were similar to Young’s modulus values where for all species, immobile joint consistently had high modulus of elasticity values compared to mobile joint and membrane of the wings as shown in Table 1.

### Amino acid composition of the wing

**Fig 11:**
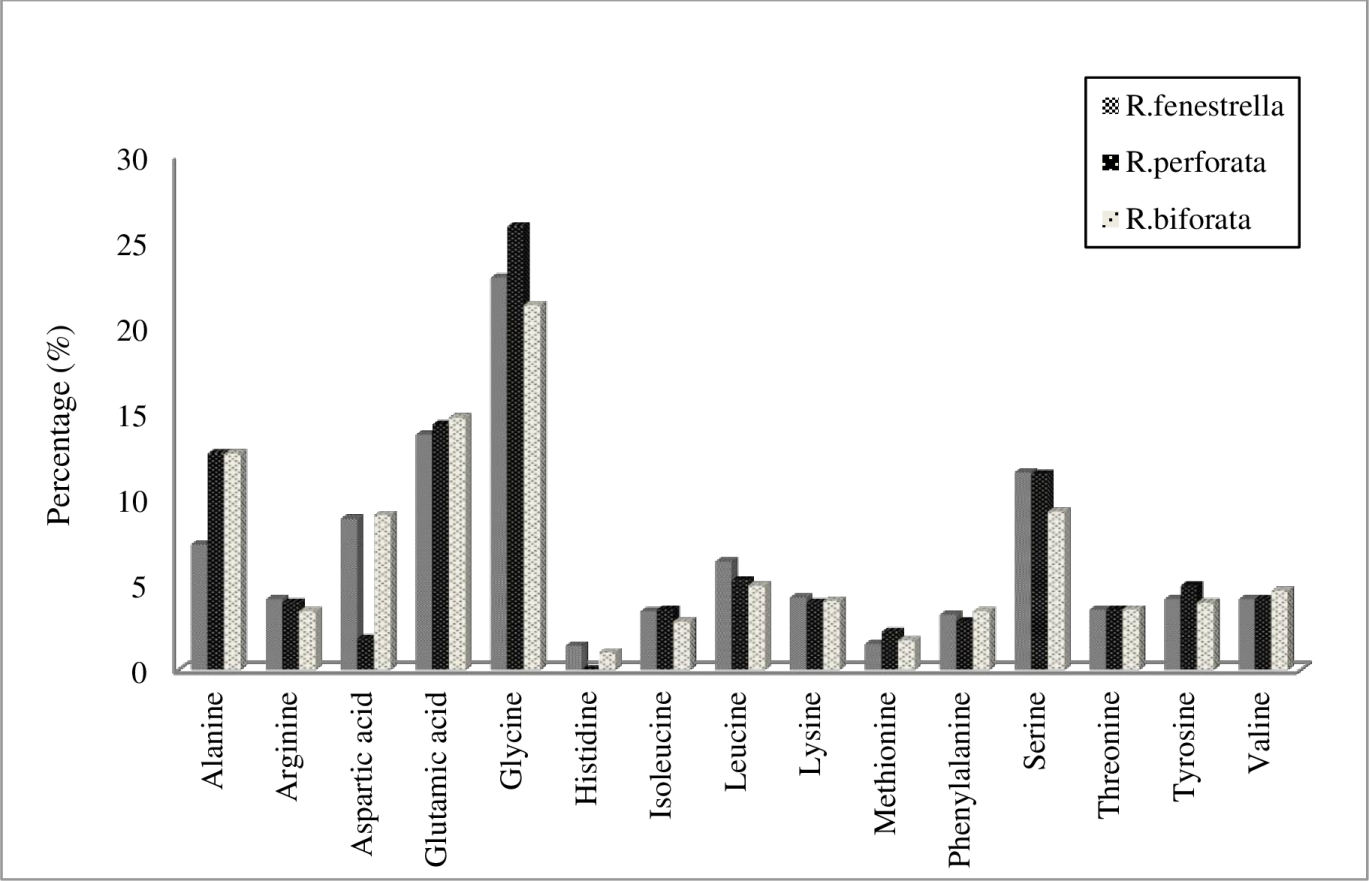
Amino acid composition in the *Rhinocypha* spp. wings.

Amino acid sequences play the main role in defining the mechanical properties at a microscopic level. Fifteen amino acids were found in our samples, with glycine, glutamic acid and alanine (except for *R*. *fenestrella*, in which alanine was replaced by serine) were found in highconcentrations (Fig 11). Glycine content ranged from 0.38 to 1.48 nmol; with 25.9% for *R*. *perforata*, 22.9% and 21.3% for *R*. *fenestrella* and *R*. *biforata* respectively.

Aspartic acid was found in variable concentrations in our samples; *R*. *fenestrella* (8.8%), *R*. *biforata* (9.0%) and least in *R*. *perforata* (1.8%), while for threonine, they shared the same value. For all species the rest of the amino acids: arginine, histidine, isoleucine, leucine, lysine, methionine, phenylalanine, threonine, tyrosine and valine were low and accounted for less than 7% of the components (see Supporting information S2 Table for data).

## Discussion

The initial scanning by SEM on the wings of *Rhinocypha* spp. had confirmed that there are two main types of joints; mobile and immobile joints as suggested by previous study [15]. According to Newman [39], there were more than six different types of cross veins. The presence of the blue-fluorescing material in the vein-joints, which was revealed by the LSCM, are known as resilin. Here, in the three species, distinct patches of resilin were found within the nodus dorsally and ventrally that are consistent with the results reported previously by Donoughe et al [17]. These unique structures are renowned to have roles in the wing torsion [21]. Moreover, resilin was found mostly in the mobile joints where cross veins meet longitudinal veins which provided elasticity and not flexibility for the twisting movements of the wing [15].

Mapping of resilin by LSCM has been summarized in Fig 5A according to nodus and major longitudinal veins by Riek and Kukalova-Peck [37] (Fig 5B). Essentially the structures of odonate wings differed in numerous ways; however, there are same sets of longitudinal veins (major longitudinal veins) which mainly conserved among them. This makes meaningful morphological comparisons between related groups. The presence of resilin at the dorsal surface with the trailing edge vein MP, is consistent with the previous study [17] when compared to dragonflies (Suborder: Anisoptera), however, among the damselflies (Suborder: Zygoptera), the longitudinal veins were categorised further by having the resilin on both dorsal and ventral surfaces. Variations in resilin patch distribution in either or both surfaces could well influence flight pattern differences in these suborders of Order: Odonata.

Additionally, the distribution of spikes was also examined in this work. Spikes were suggested to play a role in limiting free movement of the cross veins [15, 39]. In addition, spikes had a significant role in preventing material damage by inhibiting joint combinations to be too much deflected during flight [17, 40] and establishing a physical contact with the adjacent vein [10]. Previous studies initially described spike distribution concentrated at the mobile joints affecting the chord wise flexibility of the wings [15, 39]. Here, we have shown that these spikes were widespread throughout the wing surfaces and were generally located on both cross and longitudinal veins [17, 40].

The capacities of insect flights are closely related to the physical properties of the wings [41–44]. Different sections of the wings tolerate different loads, and the mechanical properties might be dissimilar because of this adaptation [45]. Currently, the development of nanoindentation techniques, for examples, [46–49] makes it conceivable to measure the modulus and hardness of insect parts. Studies on the mechanical properties, nanostructure and the basic material of the membranous wings, are still lacking [22, 50]. To fill in this gap we investigated 3 sections of the wings; membrane of the wing, mobile and immobile joints using the SEM together with the AFM to obtain topography images and to determine their elasticity. Presently, AFM is the only technique able to produce high resolution and in real time images of live microbial cell surfaces, thus it can provide information that is complementary to the information acquired from SEM [51].

The morphologies on the wing surfaces of damselflies as revealed here from SEM were patterns of series of small waves with elongated ridges was parallel to “ripple wave morphologies” reported by previous studies that may contribute to the asymmetric resistance under mechanical loading during the flight [52, 53]. Additionally, the globular nanostructures observed on the surface of the wings were similarly observed on the other species of dragonfly, *Sympetrum vulgatum* as shown by Selvakumar et al [53], thus we conclude absence of differentiation in wing surface morphologies between dragonflies and damselflies expanding from previous work [53]. Our investigations covered the whole surface of the membranous wings, the veins that were divided into mobile and immobile joints. Moreover, we further examined the amino acid sequences responsible for the mechanical properties of the wings (as discussed below).

AFM analysis of the wing with a scanned area of (1μm x 1μm) clearly showed that the surface of the wing was filled with the nanosized particles, stacked one above another. According to Selvakumar et al [53], this arrangement composed of chitin-protein and resilin, updating the results reported by Marrocco et al [54] who described that most part of the wing comprised of chitin and the joints were less stiff resilin protein. Apart from imaging capability, AFM can as well measure elastic properties of the samples via nanoindentation measurements [55–58].

The mechanical characteristics of all three *Rhinocypha* spp. wings expectedly revealed the immobile joints contained harder substances in contrast to mobile joints and the membranes of the wings. This seemed to show that the vein is the main load-bearing part of the wings [59]. Additionally, they have more flexible wing membranes, yet overall stiffer veins, when compared to the wing membranes and veins of the Anisoptera for the species; *Libellula depressa* (with modulus values: 1.5±0.5 GPa, 2.9±0.8 GPa) [60] and *Aeshna cyanea* (1.5±0.5 GPa, 2.8±0.3 GPa) [41]. This is most likely influenced by the differences in their flight performances and wing corrugations between the dragonflies and damselflies. Nevertheless, the structure of the wing that comprised of the nano globules may effect and caused the variation in measuring the stiffness of the wing. According to Kim et al [61] and Wang et al [62], in nanoindentation tests, both the reduced modulus and hardness depended on the indented area that may be affected by the surface roughness of the samples. Further Further studies by Jongerius and Lentink [63] suggested that the corrugations could enhance the stiffness and strength of the wings and resulting in a lightweight structure enhancing aerodynamics.

The insect wing is primarily composed of cuticle which is arranged in tubular, supporting veins and thin connecting membranes, with a multi-layered material of chitin microfibers fixed in a protein matrix [64]. Resilin is one of the best-known cuticle protein which is a glycine- and proline- riched protein that provides high elasticity to the cuticle on the hinge regions [35, 65, 66] and the highly dense compositions of these proteins possibly indicate the structural importance of the resilin [13].

For the production of protein, amino acids are required for structural purposes such as enzymes used for transport and storage, and also as receptor molecules [67]. Several amino acids such as; aspartic acid, glutamic acid, serine, glycine, threonine, alanine, tyrosine, valine, phenylalanine, leucine, isoleucine, proline, hydroxyproline, lysine and tryptophan are reported in the cuticle proteins and the composition of the amino acid of resilin known to be different from other structural proteins but has not been observed under the electron microscope [68]. This is due to their hydrophilicity and shared compositional characteristics with other proteins [13].

The wings of *Rhinocypha* spp. here shown to contain high amounts of glycine (results obtained from amino acid analysis) that provided elasticity to the cuticle at the hinge regions. [13, 34]. Resilin is also known to be a proline-rich protein that was not detected in our study. The existence of resilin had been confirmed by the specific fluorescence derived from resilin using a laser scanning confocal microscope. According to Rauscher et al [65], not only proline but also glycine, which was detected as the major component of resilin by the amino acid analysis in the study, can disrupt the regular protein structures. Due to the rigid conformation, proline can disrupt the secondary structure, whereas glycine, due to high flexibility, can hamper the formation of secondary structures [65, 66]. Hence, for damselflies, the function of glycine is more prominent in wing formation for flexibility.

Arginine, histidine, isoleucine, leucine, lysine, methionine, phenylalanine, threonine, tyrosine and valine were found to be low and accounted for less than 7% of the components from all the samples. An earlier study [69], reported lack of hydrophobic amino acids such as valine, leucine and isoleucine in resilin. To date, there is no research on the properties of resilin composition in dragonfly wings, although its functional importance in flight performance is known and has open up many areas of research aerodynamics, kinematics and morphological studies [39, 63, 70–73].

## Conclusion

While previous studies were on dragonflies, such as [19, 35, 41, 53, 60], this work presents the first comprehensive investigation on the structure and mechanical properties of the damselfly (*Rhinocypha* spp.) wings, combining three microscopy techniques; LSCM, SEM and AFM, together with the protein analysis. The structural analysis revealed that the flexibility of the wings varied from one area to another, and the resilin distribution pattern was the mechanism that controlled the characteristics of the wing. Furthermore, this study confirms the presence of spikes at most parts of the longitudinal veins in damselflies (Suborder: Zygoptera) which were reported to be rare in dragonflies (Suborder: Anisoptera). Additionally, the AFM images revealed resilin nanostructures of varied sizes and enabled the calculation of elasticity values at each section of the wing; membrane, mobile and immobile joints in *Rhinocypha* spp. Here we report for the first time glycine (instead of proline, as commonly described elsewhere), played a more prominent role in wing flexibility. Approaches that combine structural and mechanical studies on resilin would offer more convincing evidences for the relationship of proline -glycine structural function. While studies on silks and elastin received a lot of attention in the past decade, this has now change to focus on recombinant resilin; structure-mechanical properties of the resilin with potentially greater application in a variety of fields.

## Supporting information

S1 Table Young modulus raw data. (XLSX)

S2 Table Amino acid composition. (XLSX)

S1 File Output Young modulus. (SPV)

S2 File Proofread services. (PDF)

S3 File ITBM. (PDF)
