# Supplementary material for: Morphological and mechanical properties of flexible resilin joints on damselfly wings (Rhinocypha spp.)
Source: PLoS One. 2018 Mar 7;13(3):e0193147. doi: 10.1371/journal.pone.0193147 (PMC5841740; doi:10.1371/journal.pone.0193147)
Supplement: S2 File — (PDF) [file pone.0193147.s004.pdf]

Nesamalar_EditorialHouse
Translations/Proofreading/Editing Services
Certified from Malaysian Institute of Translation and Books (ITBM)
Facebook page: @nesamalareditorialhouse

Our ref: ANM/5/2018

Your ref: PONE-D-17-21230R1

Date: 12/01/2018

To Academic Editor PLOS ONE,

Nesamalar_EditorialHouse hereby certify that we have proofread this manuscript with the following details:

Name of Candidate: Noorhidayah Mamat
Title of Manuscript: Morphological and mechanical properties of flexible resilin joints on damselfly wings (*Rhinocypha* spp.)

This manuscript has been thoroughly checked for the English grammar and spellings. Hence, we are confident that this manuscript is fit for the final submission.

Certified,

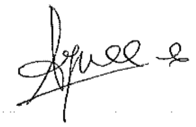

…………………………………

Agnes Nesa Malar a/p Kandan Xavier

Certified Translator/Proofreader
