# Supplementary material for: Morphological and mechanical properties of flexible resilin joints on damselfly wings (Rhinocypha spp.)
Source: PLoS One. 2018 Mar 7;13(3):e0193147. doi: 10.1371/journal.pone.0193147 (PMC5841740; doi:10.1371/journal.pone.0193147)
Supplement: S3 File — (PDF) [file pone.0193147.s005.pdf]

NO SIRI. 1278

ITBM
Institut Terjemahan & Buku Malaysia
Malaysian Institute of Translation & Books

Sijil Penyertaan

Certificate of Participation

Dengan ini disahkan bahawa
This is to certify that

AGNES NESA MALAR A/P KANDAN XAVIER

telah mengikuti
attended the

Bengkel Kerjaya dalam Penterjemahan
Career Workshop on Translation

anjuran
organised by

Institut Terjemahan & Buku Malaysia (ITBM)
Malaysian Institute of Translation & Books

telah diadakan pada
on

27 Februari - 1 Mac 2014
27 February - 1 March 2014

di
at

Hotel Permai Inn, Kuala Terengganu
Permai Inn Hotel, Kuala Terengganu

Mohd Khair bin Ngadiron
Pengarah Urusan/ *Managing Director*
Institut Terjemahan & Buku Malaysia
